# Biallelic mutations in *P4HTM* cause syndromic obesity

**DOI:** 10.2337/db22-1017

**Published:** 2023-09-01

**Authors:** Sadia Saeed, Lijiao Ning, Alaa Badreddine, Muhammad Usman Mirza, Mathilde Boissel, Roohia Khanam, Jaida Manzoor, Qasim M Janjua, Waqas I. Khan, Bénédicte Toussaint, Emmanuel Vaillant, Souhila Amanzougarene, Mehdi Derhourhi, John F Trant, Anna-Maria Siegert, Brian Y. H. Lam, Giles S.H. Yeo, Layachi Chabraoui, Asmae Touzani, Abhishek Kulkarni, I. Sadaf Farooqi, Amélie Bonnefond, Muhammad Arslan, Philippe Froguel

**Affiliations:** 1 Department of Metabolism, Digestion and Reproduction, Imperial College London, London, UK; 2 Inserm UMR 1283, CNRS UMR 8199, EGID, Institut Pasteur de Lille, Lille, France; 3 University of Lille, Lille University Hospital, Lille, France; 4 Department of Chemistry and Biochemistry, University of Windsor, Windsor ON, Canada; 5 School of Life Sciences, Forman Christian College, Lahore, Pakistan; 6 Department of Paediatric Endocrinology, Children’s Hospital, Lahore, Pakistan; 7 Department of Physiology and Biophysics, National University of Science and Technology, Sohar, Oman; 8 The Children Hospital and the Institute of Child Health, Multan, Pakistan; 9 Medical Research Council Metabolic Diseases Unit, Wellcome-MRC Institute of Metabolic Science - Metabolic Research Laboratories, University of Cambridge, Cambridge, United Kingdom; 10 Laboratory of Biochemistry and Molecular Biology -Faculty of Medicine and Pharmacy - University V Mohamed Rabat, Moroccoo; 11 Children’s Hospital of Rabat and Laboratory of Biochemistry and Molecular Biology - Faculty of Medicine and Pharmacy - University V Mohamed Rabat -Moroccoo; 12 Department of Paediatric Endocrinology, Sir HN Reliance Foundation & SRCC Children’s Hospital, Mumbai, India

## Abstract

We previously demonstrated that 50% of children with obesity from consanguineous families from Pakistan carried pathogenic variants in known monogenic obesity genes. Here, we have discovered a novel monogenetic recessive form of severe childhood obesity, using an in-house computational staged approach. This included analysis of whole-exome sequencing data of 366 children with severe obesity, 1,000 individuals of the Pakistani PROMIS study, and 200K participants of the UK Biobank, to prioritize genes harbouring rare homozygous variants with putative effect on human obesity. We identified five rare or novel homozygous missense mutations predicted deleterious in five consanguineous families in *P4HTM* encoding Prolyl 4-Hydroxylase Transmembrane (P4H-TM). We further found two additional homozygous missense mutations in children with severe obesity of Indian and Moroccan origin. Molecular dynamics simulation suggested that these mutations destabilized the active conformation of the substrate binding domain. Most carriers also presented with hypotonia, cognitive impairment and/or developmental delay. Three of the five probands died of pneumonia during the ~2 years of the follow up. *P4HTM* deficiency is a novel form of syndromic obesity affecting 1.5% of our children with obesity associated with high mortality. P4H-TM is a hypoxia inducible factor that is necessary for survival and adaptation under oxygen deprivation but the role of this pathway in energy homeostasis and obesity pathophysiology remains to be elucidated.

## Introduction

Previously, our genetic studies on children with severe obesity from consanguineous families of Pakistan (severely obese Pakistani population/SOPP cohort), enabled a successful genetic diagnosis in ~50% of obese participants (1–5). This predominantly includes identification of pathogenic mutations in genes that play a key role in the hypothalamic leptin-melanocortin pathway (e.g. *LEP, LEPR* and *MC4R*), regulating appetite and energy homeostasis. Early-onset, severe obesity accompanied with severe hyperphagia are the most common phenotypes caused by these mutations.

There are also several well-known pleiotropic monogenic obesity syndromes where obesity is one of a number of clinical anomalies often including intellectual disability, structural abnormalities, dysmorphic features, vision and hearing impairments, maladaptive behaviour, and organ- and cell-specific anomalies, as observed in Bardet-Biedl, Alstrom, Carpenter and Cohen syndromes (6), amongst others. Our endeavour of unravelling genetic causality in SOPP also led to the identification of probands with these obesity syndromes (7) and also uncovered that bi-allelic mutations in *ADCY3* are a novel genetic cause of syndromic obesity (8).

Aiming at a further investigation of the genetic basis of severe obesity and to identify additional causes of obesity in undiagnosed cases from SOPP, we developed and employed an in-house systematic analysis of the sequencing data using MiST method (9) in combination with rigorous and step-wise data filtration. Additionally, we carried out molecular dynamics (MD) simulations to determine consequences of the mutations on the encoded proteins’ stability and conformation. As a result, we have identified several patients with severe obesity carrying homozygous, deleterious, missense mutations in *P4HTM* encoding the enzyme prolyl 4-hydroxylase transmembrane (P4H-TM), an atypical member of the hypoxia inducible factors prolyl 4-hydroxylases (HIF-P4Hs), that are necessary for adaptation under reduced oxygen supply. This is the only member of HIF-P4Hs that predominantly expresses in brain but the mechanism by which P4H-TM causes obesity is still unknown thus opening new avenues of research in the physiology of energy balance.

## Materials and Methods

### Genetic and Statistical analysis

Initially, all probands with severe obesity from SOPP (*n*=456) were systematically screened for mutations in *LEP* and *MC4R* through Sanger sequencing. The probands found negative for pathogenic or likely pathogenic mutations in these two genes, were further analysed either through conventional whole-exome sequencing or augmented whole exome sequencing (CoDE-seq) described in detail elsewhere (10; 11).

Analysis of exome data was carried out stage-wise: **Stage I** All the genes known to be linked with monogenic obesity including syndromic obesity, were searched for pathogenic or likely pathogenic mutations by following the rules and guidelines of the American College of Medical Genetics and Genomics (Supplementary Table 1) (12). **Stage II** Gene-centric analysis using the MiST method was performed (9) on exome data from severely obese subjects with yet undiagnosed genetic causality, in SOPP (*n*=366) and 1,003 normal-weight subjects from PROMIS (Pakistan Risk of Myocardial Infarction Study) cohort. The MiST method was first published in 2013 (9) and since then has been used in several studies that demonstrate that MiST performs best with regard to its statistical power across a range of architectures (13–16).

**Figure 1:**
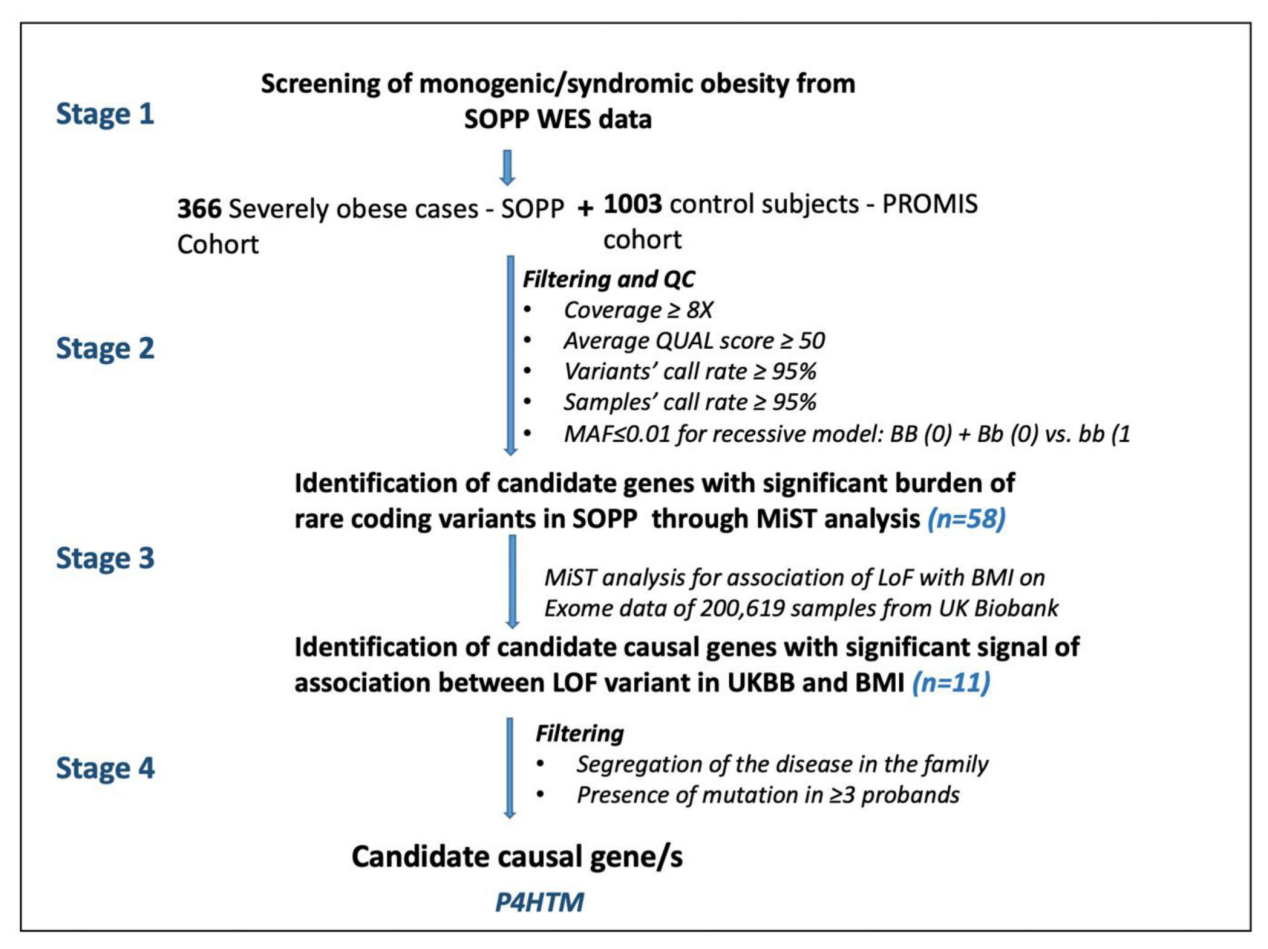
Schematic presentation of stepwise analyses and filtration strategy used in this study.

Participants in both groups belong to the same geographical region allowing for a better comparison, to identify genes harbouring a significant burden of rare (*i.e*. with a minor allele frequency below 1%), homozygous and potentially deleterious (according to SIFT and PolyPhen) variants among the cases with obesity. In **Stage III**, the genes identified with a significant p-value at **Stage II** (*i.e. P*_π_ < 0.05) were investigated against 200K exome data from UK Biobank (Application #67575), for assessment of association between null variants (*i.e*. nonsense, frameshift, canonical ±1 or 2 splice sites, initiation codon) per gene and body mass index (BMI) using MiST. Our statistical analyses were adjusted for age, gender and the first 5 genetic principal components, to take into account any potential ethnicity confounds in the cohort. The details of the MiST analysis have been provided elsewhere (17). In the **Stage IV**, only the genes with mutations in 3 or more unrelated families were considered as to be likely involved in human obesity. Finally, in **Stage V**, literature survey and further clinical investigation of the affected probands was carried out (Figure 1).

### Biochemical analysis

Metabolic markers including leptin, insulin and cortisol were determined using commercially available ELISA kits (Monobind, Lake Forest).

### Molecular modelling

The molecular modelling was carried out for four of the seven mutations (p.Glu155Lys, p.Val297Met, p.Val316Ile and p.Gly433Ala), where crystal structural information was available. Here the dynamic and structural consequences of the P4HTM mutations on substrate binding were assessed through homology modelling followed by molecular dynamics (MD) simulations. The detailed procedure is given in Supplementary Information.

### *P4HTM* expression in murine and human hypothalamus

Recently we generated an integrated single-cell atlas, namely HypoMap, of the murine hypothalamus. It consists of 384,924 cells collected from 18 different single-cell studies, covering various regions of the hypothalamus (18). The publicly available Seurat data object was used to assess the expression of *P4htm* in cells captured within the atlas.

To examine the expression for *P4HTM* in human hypothalamus, we extracted the hypothalamic cells from a single-nucleus sequencing dataset from adult human brains (19), available via the Neuroscience Multi-omics Archive (NeMO, RRID:SCR_016152). Briefly, the count data from 134,471 nuclei labelled ‘hypothalamus’ from ROIGroupCoarse was extracted from the original loom file. The count table was then imported into Seurat 4.1.1 for log-normalisation; variable feature selection (3000 genes); data reduction via principal component analysis (PCA, 90 PCs); and uniform manifold approximation projection (UMAP using PCs 1:50) and used for data visualisation.

## Results

The stepwise analysis and rigorous filtration criteria (Figure 1) resulted in the identification of only one gene putatively involved in monogenic obesity: *P4HTM* (encoding Prolyl 4-Hydroxylase, Transmembrane). Through gene-centric analysis the burden of rare, homozygous, and potentially deleterious variants in the *P4HTM* was significantly associated with obesity risk (*P*_π_ = 0.013; π_hat =14; 95% CI_2.5= -26; *P*_τ_ =1.0). In the next step (*i.e*. Stage III) using the 200K exome data from UK Biobank, we assessed the association between null variants (Supplementary Table 2) in *P4HTM* and adiposity. We found that these null variants were significantly associated with higher BMI levels (*P*_π_ = 0.017; π_hat =1.4; 95% CI 0.25–2.5; *P*_τ_ = 0.89). Notably, all the null variants that were detected in the UK biobank were heterozygous.

A total of seven mutations were identified In *P4HTM* in seven unrelated probands (all males), from consanguineous families. In the SOPP cohort, five different homozygous missense mutations (p.Pro45Leu, p.Val297Met, p.Val316Ile, p.Gly433Ala and p.Arg509Pro) were identified in 5 unrelated probands (Table 1; Supplementary Figure 1). A further screening of *P4HTM* in other cohorts identified 2 additional subjects with severe obesity carrying homozygous missense mutations (p.Gly116Asp and p.Glu155Lys), from consanguineous families of Indian and Moroccan origin, respectively. All the mutations identified and reported here are either novel or with a very rare minor allele frequency (≤ 0.0002). Of these, p.Glu155Lys is present in EF-hand domain whereas p.Val316Ile, p.Gly433Ala and p.Arg509Pro are in the prolyl 4-hydroxylase domain (Table 1; Supplementary Figure 1).

The seven mutation carriers presented severe, early-onset obesity between 0.3 to 3 years with a BMI SDS for age ≥ 4. In addition to obesity, the carriers presented with hypotonia, developmental delay and cognitive impairment. Also, a variable set of other abnormalities was also observed in individual cases. Three of 5 probands from SOPP died from respiratory infection during the first ~2 years of the follow up (Supplementary Figures 1,2, Table 2).

**Figure 2:**
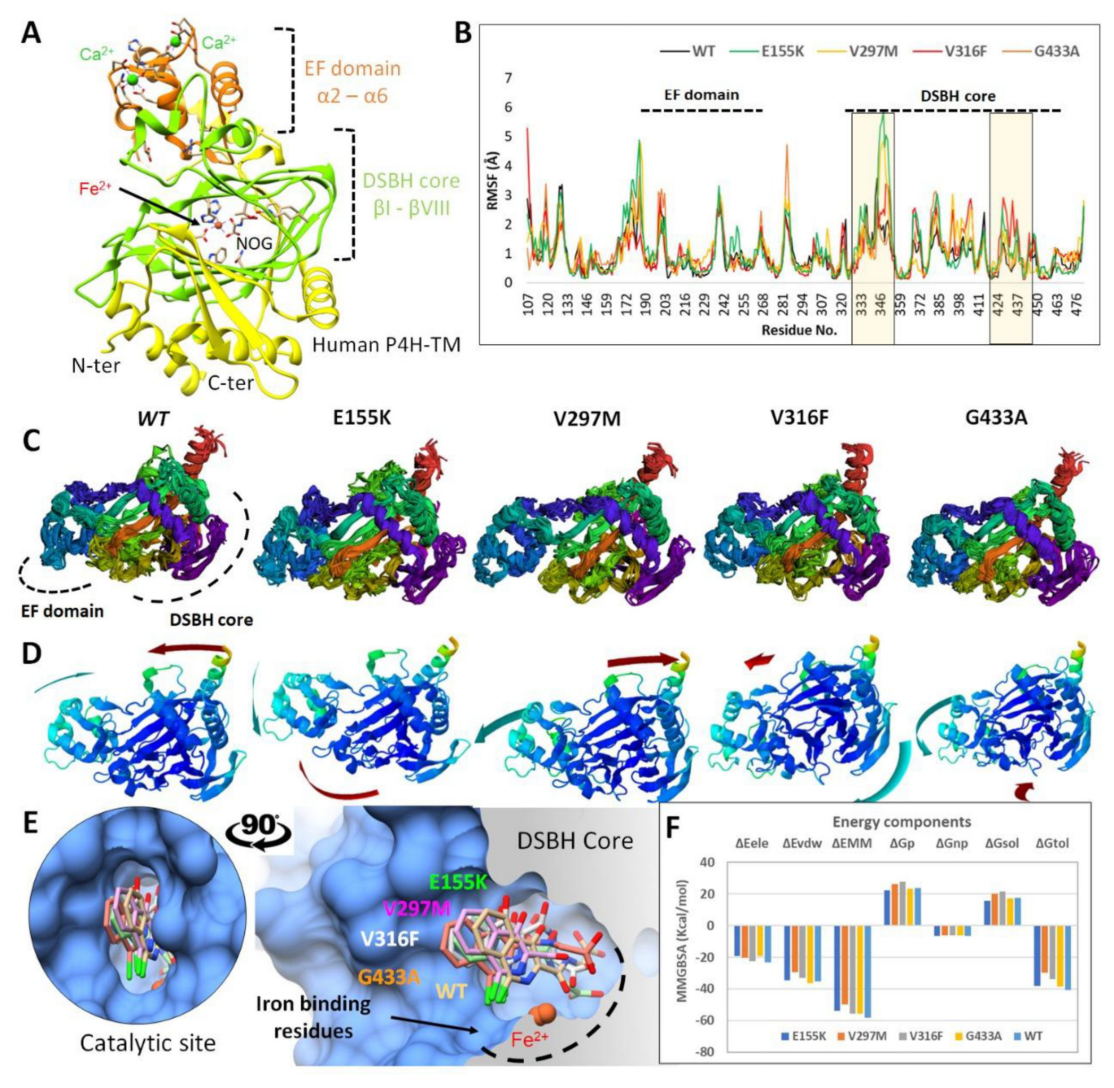
Structural interpretation of *P4HTM* missense variants. **A)** The *P4HTM* structure is presented as a secondary structure topology, where the EF domain is shown in orange, the DSBH core in green, and the rest of the protein in yellow. **B)** Root-mean-square-deviation of mutants and wild-type (black) obtained over a period of 100 ns. The individual domains are mentioned, while iron-binding residues are highlighted in the yellow section. **C)** The MD simulated conformations (10 snapshots after every 20 ns) of mutants and wild type. Different regions of *P4HTM* are colored to increase the visibility of backbone deviations. **D)** The movement of domains (EF and DSBH) are displayed during MD simulations. **E)** Superimposition of MD simulated conformations of FG-2216 (sticks representation in distinct colour) in the active site of mutants and wild-type. Fe^2+^ is positioned inside the active site of *P4HTM* (molecular surface representation). **F)** Molecular mechanics generalized born surface area (MM-GBSA) binding free energies (kcal/mol) for mutants and wild-type/FG-2216 complex.

The prediction of the stability of the protein was carried out in four out of seven mutations where crystal structural information was available. The quantitative stability changes (ΔΔG) upon mutations indicated a destabilizing effect and revealed an increase in flexibility (Supplementary Table 3). Through extensive molecular dynamics simulations, mutants showed fluctuations in both catalytic domains, EF hand (residues 190 - 290) domain, and the double-stranded β-helix (DSBH) fold (residues 310 - 460) (Figure 2). Overall, the structural analysis revealed domain movements compared to its *wt-P4HTM*. Details of the structural interpretation of mutations are provided in the Supplementary Information.

Finally, using ‘HypoMap’, an integrated reference atlas of the murine hypothalamus (18), we found that *P4HTM* is expressed ubiquitously in all regions of the hypothalamus, and in all cell-types, including neurons, oligodendrocytes, astrocytes and tanycytes (Supplementary Figure 3). Similar expression of P4HTM was observed in the hypothalamic cells from a single-nucleus sequencing dataset from adult human brains. This broad expression profile is consistent with the observed complex phenotype, beyond severe obesity, of the patients (Supplementary Figure 5). Non-fasting serum levels of metabolic hormones, leptin, insulin and cortisol, determined in the five affected patients from the SOPP were within the normal range except in one proband that presented hyperinsulinemia (Table 2).

## Discussion

We report seven paediatric patients with severe obesity carrying rare or novel bi-allelic missense mutations in *P4HTM*, representing a form of monogenic syndromic obesity, following a stepwise gene-centric analysis. The affected subjects were previously found negative for all known obesity genes. The phenotypes of all the affected subjects, in addition to obesity, included hypotonia, intellectual disability, and developmental delay. Importantly, these neurologic characteristics are consistent with the recently proposed HIDEA syndrome (acronym for **h**ypotonia, **i**ntellectual **d**isability, and **e**ye **a**bnormalities). This syndrome was first described in 2014 in a single Finnish family carrying pathogenic mutations in three genes - transketolase (*TKT*), prolyl 4-hydroxylase transmembrane (*P4HTM*), and ubiquitin specific peptidase 4 (*USP4*) (20). HIDEA syndrome was later suggested to be only caused by biallelic pathogenic mutations in *P4HTM*, and associates with hypotonia, hypoventilation, intellectual disability, dysautonomia, developmental delay and eye abnormalities (21). To date, 10 families have been reported with homozygous or compound heterozygous mutations in *P4HTM* (20–23). Obesity phenotype was not emphasized in any of the reports, but our secondary assessment of the published phenotypes revealed that in 2 families, mutation carriers presented obesity (21; 23).

It is noteworthy that all *P4HTM* mutations found in the SOPP cohort are missense, which is also the case for the three previously published individuals with severe obesity from the same family(23). On the contrary, all the other families showing severe neurological phenotypes that characterize HIDEA but lacking obesity (with the exception of one individual), have loss-of-function mutations (i.e. stop-gain, frame-shift and splice site). It is possible that this phenotypic heterogeneity reflects an allelic heterogeneity responsible for variable impairment of brain function, that affects or not the central control of appetite and/or energy balance.

*P4HTM* encodes the enzyme P4H-TM, an atypical member of the hypoxia inducible factors prolyl 4-hydroxylases (HIF-P4Hs) that are necessary for survival and adaptation under reduced oxygen supply and oxygen deprivation (24). Four members of the HIF-P4H family have so far been identified (25). In a scenario of normal oxygen supply - normaxia, the HIF-P4Hs get activated resulting in post-translational hydroxylation of HIF-α by converting prolines to 4-hydroxyprolines leading to its degradation. In the event when cells suffer from hypoxia, the HIF-α related hydroxylation reactions mediated by the P4Hs are suppressed resulting in HIF-α accumulation and its dimerization with HIF-β and migration into the nucleus for activation of several target genes responsible for maintaining oxygen homeostasis (26). Unlike other P4Hs members, P4H-TM is a transmembrane enzyme with a high degree of expression in the brain, predominantly in the hippocampus, amygdala and hypothalamic regions (27). However, its physiological function in the brain is yet unknown (28; 29). Our data suggest that genetic disruption of *P4HTM* also causes childhood obesity, possibly through dysregulation of appetite.

The very high mortality at a very young age of the children with *P4HTM* mutations, of the SOPP cohort (7 documented deaths including 3 probands and 4 family mutation carriers) is an ongoing tragedy. Furthermore, the deaths are apparently not related to central defects but possibly to lung hypoventilation following pneumonia, and possibly associated with defective immunity (as reported in leptin deficient children).

In summary, pathogenic mutations in *P4HTM* cause, in the SOPP cohort, severe obesity associated with neurologic features of the HIDEA syndrome in 1.5% of our probands. It is not restricted to Pakistan as we identified two other patients from consanguineous families of Indian and Moroccan origin. The severity of this obesity associated syndrome with a high risk of mortality, highlights the advisability of screening *P4HTM* in young patients with severe obesity that also present (or express) hypotonia and intellectual disability/developmental delay, for a timely and appropriate management and care. Beyond linking *P4HTM* to obesity, we have demonstrated the power of stepwise statistical analysis using MiST followed by stringent filtration steps in bringing to surface new genes that are also associated with obesity.

## Supplementary Material

Supplementary Material

## Figures and Tables

**Table 1:** Homozygous mutations identified in *P4HTM* (NM_177938.2) among severely obese children from consanguineous populations.

ID	Mutation	PolyPhen	SIFT	Origin (study)	MAF overall (in GnomAD)	MAF SA in GnomAD)
Proband I	c.1298G>C; p.Gly433Ala	Probably Damaging	Damaging	Pakistan (SOPP)	NA	NA
Proband II	c.946G>A; p.Val316Ile	Probably Damaging	Damaging	Pakistan (SOPP)	0.000003	0.000
Proband III	c.1526G>C; p.Arg509Pro	Probably Damaging	Damaging	Pakistan (SOPP)	NA	NA
Proband IV	c.889G>A; p.Val297Met	Probably Damaging	Damaging	Pakistan (SOPP)	0.00001	0.00013
Proband V	c.134C>T; p.Pro45Leu	Possibly Damaging	Damaging	Pakistan (SOPP)	0.0002	0.0021
Proband VI	c.347G>A; p.Gly116Asp	Probably Damaging	Damaging	Indian (GOOS)	0.000005	0.00003
Proband VII	c.463G>A; p.Glu155Lys	Probably Damaging	Damaging	Morocco	NA	NA

MAF SA: Minor Allele Frequency South Asian, NA: Not available.

**Table 2:** Clinical profile of severely obese children identified with balletic mutations in *P4HTM*.

ID	Proband I	Proband II	Proband III	Proband IV	Proband	Proband VI	Proband VII
**Age at first recruitment (Years)**	4.5	0.9	5.0	3.9	9.6	3.0	4.0
**Sex M/F**	M	M	M	M	M	M	M
**Age of obesity onset (Years)**	0.9	0.3	1.0	0.6	3.0	NA	NA
**SDS (BMI for age)**	6.8	6.7	6.8	8.0	5.7	NA	NA
**Alive/deceased**	Deceased (5.2 years of age due to severe pneumonia)	Deceased (1.5 years of age due to pneumonia and high-grade fever)	Deceased (6 years of age; cause of death unknown)	Alive	Alive	Alive	Alive
**Leptin (ng/ml)**	3	18	10	16	12	NA	41.9
**Insulin (ulU/ml)**	26	19	122	5	12	NA	NA
**Cortisol (ug/dl)**	11	7	11	10	6	NA	NA
**Intellectual disability**	Yes	Yes	Yes	Yes	No	Yes	Yes
**Hypotonia**	Yes	Yes	Yes	Yes	Yes	Yes	Yes
**Deaths in family**	The sisters at the age of 2 and 3.5 years and one cousin at age of 1.5 died as the result of kidney dysfunction (proteinuria)	His elder brother who shared the similar clinical phenotype died at the age of 2 years due to pneumonia	NR	NR	NR	NR	NR
**Birth by caesarean section**	Yes	Yes	No	Yes	No	NA	NA
**Additional features**	Polydipsia and polyuria, eye abnormalities including astigmatism, reported stubborn and aggressive in temperament	Suffered hepatomegaly, asthma and abdominal pain	Delayed milestones, aggressive behaviour, self-mutilation/self harm, neonatal hypotonia, Thalassemia	Scoliosis, abnormal gait (needs support for walking, pneumonia twice but recovered, aggressive and stubborn	Asthma, breathing issue, sleep apnea	Epilepsy, hypoventilation, developme ntal and cognitive impairment, autonomic dysfunction	Hearing issues, cryptorchidism

F: Female; M: Male; NR: Not reported; SDS: Standard Deviation Score.

## Data Availability

The data sets generated during the current study are available upon reasonable request. No applicable resources were generated during the current study.
