# Supplementary material for: Biallelic mutations in P4HTM cause syndromic obesity
Source: Diabetes. Author manuscript; Available in PMC 2025 Mar 14. (PMC7617486; doi:10.2337/db22-1017)
Supplement: Supplementary Material [file EMS203462-supplement-Supplementary_Material.pdf]

SUPPLEMENTARY INFORMATION

Biallelic mutations in *P4HTM* cause syndromic obesity

Sadia Saeed[1,2,3§], Lijiao Ning[2,3], Alaa Badreddine[2,3], Muhammad Usman Mirza[4], Mathilde Boissel[1,2,3], Roohia Khanam[5], Jaida Manzoor[6], Qasim M Janjua[7], Waqas I. Khan[8], Bénédicte Toussaint[2,3], Emmanuel Vaillant[2,3], Souhila Amanzougarene[2,3], Mehdi Derhourhi[2,3], John F Trant[4], Anna-Maria Siegert[9], Brian Y. H. Lam[9], Giles S.H. Yeo[9], Layachi Chabraoui[10], Asmae Touzani[11], Abhishek Kulkarni[12], I. Sadaf Farooqi[9], Amélie Bonnefond[1,2,3*], Muhammad Arslan[5*] and Philippe Froguel[1,2,3*§]

[1]Department of Metabolism, Digestion and Reproduction, Imperial College London, London, UK; [2]Inserm UMR 1283, CNRS UMR 8199, EGID, Institut Pasteur de Lille, Lille, France, [3]University of Lille, Lille University Hospital, Lille, France; [4] Department of Chemistry and Biochemistry, University of Windsor, Windsor ON, Canada, [5]School of Life Sciences, Forman Christian College, Lahore, Pakistan, [6]Department of Paediatric Endocrinology, Children's Hospital, Lahore, Pakistan, [7]Department of Physiology and Biophysics, National University of Science and Technology, Sohar, Oman, [8]The Children Hospital and the Institute of Child Health, Multan, Pakistan,[9]Medical Research Council Metabolic Diseases Unit, Wellcome-MRC Institute of Metabolic Science - Metabolic Research Laboratories, University of Cambridge, Cambridge, United Kingdom, [10]Laboratory of Biochemistry and Molecular Biology -Faculy of Medicin and Pharmacy -University V Mohamed Rabat, Moroccoo, [11]Children 's Hospital of Rabat and Laboratory of Biochemistry and Molecular Biology - Faculy of Medicine and Pharmacy -University V Mohamed Rabat -Morocco, [12]Department of Paediatric Endocrinology, Sir HN Reliance Foundation & SRCC Children's Hospital, Mumbai, India

*These authors contributed equally to the paper

[§]Corresponding authors: Sadia Saeed, s.saeed08@imperial.ac.uk; Philippe Froguel, p.froguel@imperial.ac.uk

Supplementary Table 1

Supplementary Table 1. List of genes linked with monogenic obesity.

Gene	Name	Location	Mendelian disorder	NM transcript
ALMS1	ALMS1, centrosome and basal body associated protein	2p13.1	Obesity & syndromic features	NM_015120.4
ADCY3	Adenylate Cyclase 3	2p23.3	Obesity & syndromic features	NM_004036.4
BBS1	Bardet-Biedl syndrome 1	11q13.2	Obesity & syndromic features	NM_024649.4
BBS10	Bardet-Biedl syndrome 10	12q21.2	Obesity & syndromic features	NM_024685.3
BBS12	Bardet-Biedl syndrome 12	4q27	Obesity & syndromic features	NM_152618.2
BBS2	Bardet-Biedl syndrome 2	16q13	Obesity & syndromic features	NM_031885.3
BBS4	Bardet-Biedl syndrome 4	15q24.1	Obesity & syndromic features	NM_033028.4
BBS5	Bardet-Biedl syndrome 5	2q31.1	Obesity & syndromic features	NM_152384.2
BBS7	Bardet-Biedl syndrome 7	4q27	Obesity & syndromic features	NM_176824.2
BBS9	Bardet-Biedl syndrome 9	7p14.3	Obesity & syndromic features	NM_001033604.1
BDNF	brain derived neurotrophic factor	11p14.1	Obesity & syndromic features	NM_170735.5
CEP19	centrosomal protein 19	3q29	Obesity & syndromic features	NM_032898.4
CEP290	centrosomal protein 290	12q21.32	Obesity & syndromic features	NM_025114.3
LEP	leptin	7q32.1	Obesity	NM_000230.2
LEPR	leptin receptor	1p31.3	Obesity	NM_002303.5
MC4R	melanocortin 4 receptor	18q21.32	Obesity	NM_005912.2
MKKS	McKusick-Kaufman syndrome	20p12.2	Obesity & syndromic features	NM_018848.3
MKS1	Meckel syndrome, type 1	17q22	Obesity & syndromic features	NM_017777.3
MRAP2	melanocortin 2 receptor accessory protein 2	6q14.2	Obesity	NM_138409.2

NTRK2	neurotrophic receptor tyrosine kinase 2	9q21.33	Obesity & syndromic features	NM_006180.4
PCSK1	Proprotein convertase subtilisin/kexin type 1	5q15	Obesity	NM_000439.4
POMC	proopiomelanocortin	2p23.3	Obesity	NM_001035256.1
TUB	tubby bipartite transcription factor	11p15.4	Obesity & syndromic features	NM_003320.4
VPS13B	vacuolar protein sorting 13 homolog B	8q22.2	Intellectual disability & obesity	NM_017890.4

Supplementary Table 2 Null mutations in *P4HTM* (NM_177938.2) identified from the 200K exome data from UK Biobank and analyzed in the study.

**Mutation**	**Consequence**	**gnomAD_SAS**
c.1074_1075insT; p.Gln359SerfsTer85	frameshift	6.533e-05
c.1208G>A; p.Trp403*	stop_gained	0
c.1231del; p.Cys411AlafsTer11	frameshift	0
c.1246del; p.Leu416CysfsTer6	frameshift	7.712e-05
c.1246dup; p.Leu416ProfsTer28	frameshift	0.0001542
c.1247del; p.Leu416Argfs*6	frameshift	3.614e-05
c.1348_1349del; p.Thr450Glnfs*9	splice_acceptor	-
c.1348-1G>A; p.Gln476*	stop_gained	-
c.1446G>A; p.Trp482*	stop_gained	0
c.1511dup; p.Cys505Leufs*16	frameshift	-
c.1523_1524insCAGGCAGCCCCCTGGTCAC; p.Gly510Glnf*17	frameshift	-
c.153del; p.Lys51Asnfs*14	frameshift	-
c.1627_1628del; p.Gly543Hisfs*85	frameshift	-
c.1654_1655del; p.Leu552Glyfs*76	frameshift	-
c.1666dup; p.Tyr556Leufs*73	frameshift	-
c.1674del; p.Asp558Glufs*36	frameshift	-
c.1A>C; p.Met1?	start_lost	-
c.286dup; p.Gln96Profs*29	frameshift	-
c.2dup; p.Met1?	start_lost	-
c.343G>T; p.Glu115*	stop_gained	0
c.436+2del	splice_donor	-
c.571del; p.Leu191Trpfs*32	frameshift	-
c.611_612insGTGC; p.His204Glnfs*7	frameshift	-
c.637C>T; p.Gln213*	stop_gained	-

c.659G>A; p.Trp220*	stop_gained	0
c.724G>A; p.Gly242Arg	Splice-site	0
c.72G>A; p.Trp24*	stop_gained	0
c.740del; p.Gln247Argfs*15	frameshift	-
c.780del; p.Tyr260*	stop_gained	-
c.881_887+1dup	frameshift	-
c.952C>T; p.Arg318*	stop_gained	0

gnomAD_SAS: gnomAD minor allele frequency in South Asian Population

Supplementary Table 3. Estimated protein structure quantitative stability changes due to biallelic mutations in *P4HTM*.

Mutated models	DUET	ENCoM	
	**Consensus prediction from mCSM and SDM (ΔΔG kcal.mol^{-1})**	**Vibrational Entropy Energy (ΔΔS vib kcal.mol^{-1}.K^{-1})**	**Thermal stability (ΔΔG kcal.mol^{-1})**
p.Glu155Lys	-0.42 (destabilizing)	0.523 (increase in flexibility)	-0.21 (destabilizing)
p.Val297Met	-2.14 (destabilizing)	1.53 (increase in flexibility)	-1.297 (destabilizing)
p.Val316Ile	-1.04 (destabilizing)	0.631 (increase in flexibility)	-0.256 (destabilizing)
p.Gly433Ala	-0.60 (destabilizing)	0.231 (increase in flexibility)	-0.756 (destabilizing)

Note: DUET; a web server for an integrated computational approach that combines two complementary approaches in a consensus prediction. ENCoM; a coarse-grained normal mode analysis method to predict the effect of mutations on thermostability and dynamics of protein structure. ΔΔG; Gibbs Free Energy and negative values indicate destabilizing mutations. ΔΔSvib; vibrational entropy changes, while negative value represents the rigidification of the protein structure and positive value represents gain in flexibility

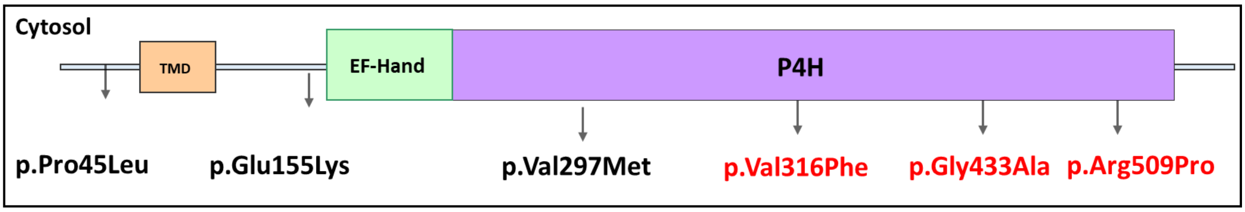

Supplementary Figure 1. Schematic presentation of *P4HTM* indicating the location of point mutations identified in severely obese children. Mutations in red indicate carriers in which early deaths have been reported. ***P4H,*** prolyl 4-hydroxylase domain; ***TMD,*** transmembrane domain

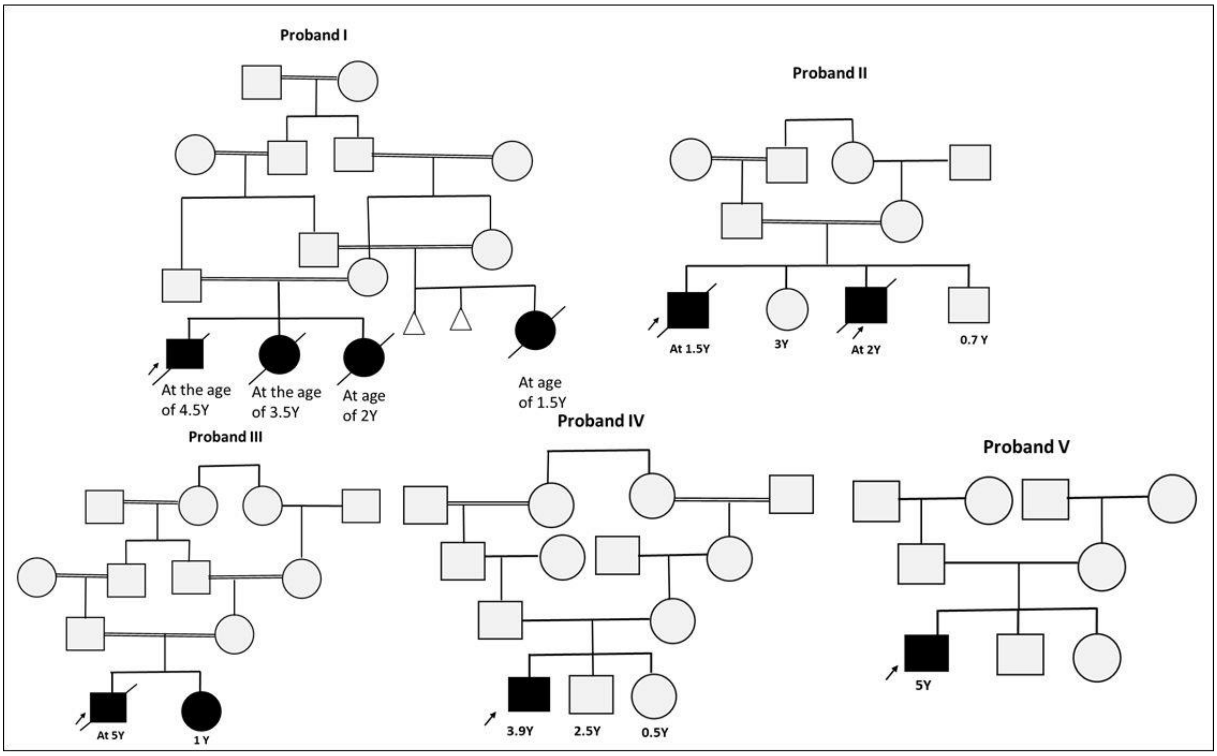

Supplementary Figure 2. Family tree of probands with mutations in the *P4HTM* gene linked with monogenic syndromic form of severe obesity.

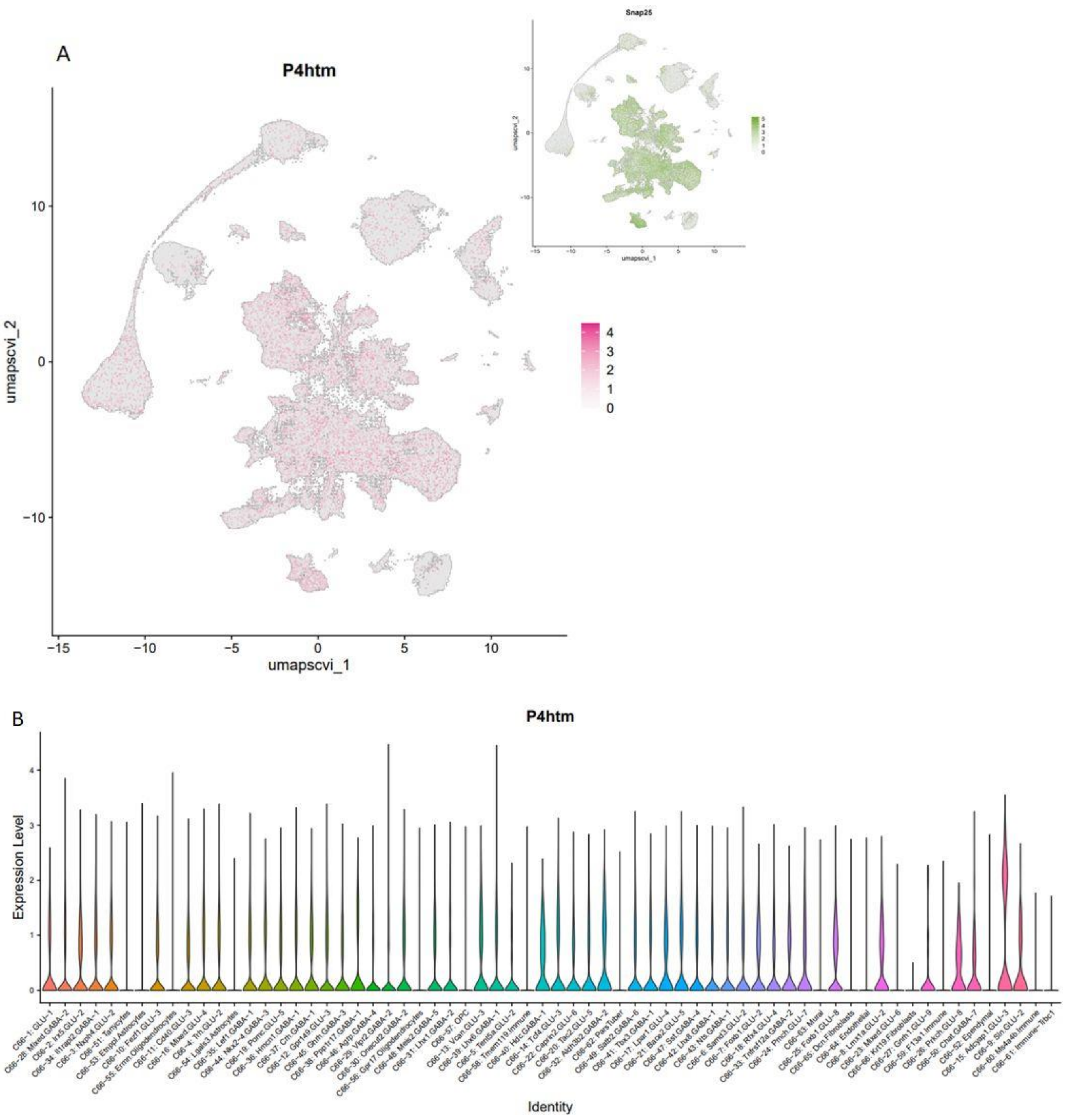

Supplementary Figure 3. HypoMap - unified hypothalamic cell atlas showing wide expression of P4htm. (A) A UMAP dimensional reduction plot (inset shows the expression of neuronal marker Snap25); and (B) violin plot showing the expression of P4htm in the majority of neuronal populations. Color corresponds to log2-normalized expression level.

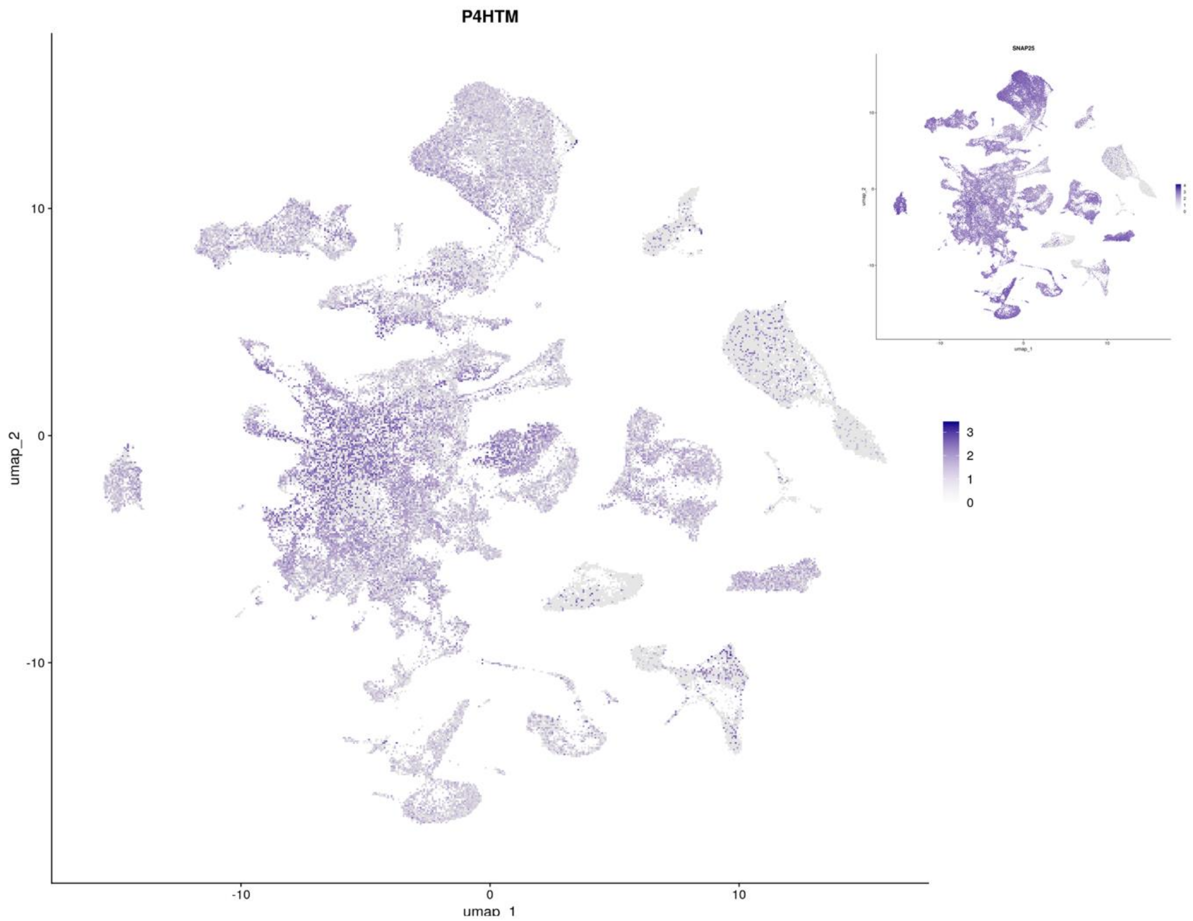

Supplementary Figure 4: The expression for *P4HTM* in human hypothalamic cells from a single-nucleus sequencing dataset from adult human brains (inset shows the expression of neuronal marker Snap25).

Supplementary Information

Molecular Modelling:

To understand the dynamic consequences of mutations on *P4HTM,* 3D coordinates of the x-ray crystal structure of *P4HTM* were obtained from the Protein Data Bank (PDB: *6TP5*)[1]. The mutant structures were generated through amino acid substitution, based on sidechain torsion (Chi) and probability values using the rotamers tool of UCSF chimera[2]. Mutational effects on protein stability were predicted using the DUET server[3,4] and thermal stability (ΔΔG) resulting from vibrational entropy changes (ΔΔS) using the Elastic Network Contact Model (ENCoM) server [5]. The mutational consequences in substrate binding site of *P4HTM* were also investigated through molecular docking of orally active HIF-prolyl hydroxylase inhibitor, FG-2216 [1], inside the active site of *P4HTM*. Molecular docking was performed by AutoDock Vina using a standard protocol as described previously[6]. The impact of the mutation on the overall structural dynamics of *P4HTM* and binding conformations of FG-2216 in mutants compared to its wildtype (*wt*) was analyzed using MD simulations. The MD simulation protocol was performed in two steps: 20ns MD simulation to refine and optimize mutants, and a second 100 ns simulation to observe the residual fluctuations of *P4HTM* with or without reported mutations. All simulations were carried out by AMBER 18[7] using the protocol previously described by us and others[8,9]. Briefly, the Antechamber package of AmberTools was utilized, and parameters were obtained from the GAFF force field (GAFF)[10]. Since *P4HTM* holds Fe^{2+} in the active site, the force field parameters of Fe^{2+} were calculated using MTK^{++}/MCPB facility[11] of AmberTools. Coordinate trajectories were collected every 2 picoseconds for the complete 100ns production run, and the CPPTRAJ module of AMBER18 was utilized to analyze the root-mean-square fluctuations[12]. The molecular mechanics-generalized born

surface area (MM-GBSA) was calculated using 1000 snapshots extracted from the trajectory. The complexes were analyzed using Chimera 1.13[13].

Structural interpretation of mutations

The crystal structure of *P4HTM is* composed of two well-defined domains: the catalytic domain with the EF-hand (residues 190 - 290) domain and the double-stranded β-helix (DSBH) fold (residues 310 - 460) (**Figure 1A**). All reported mutations were located in the core domains, p.E155K in the EF domain, whereas p.V297M, p.V316F and p.G433A were present in the DSBH core region. The quantitative stability changes (ΔΔG) upon mutations were predicted using the DUET program. All four mutations, p.E155K, p.V297M, p.V316F, and p.G433A in *P4HTM* were predicted as destabilizing with negative free energy change (ΔΔG) values of -0.42, -2.14, -1.04, and -0.60 kcal/mol, respectively. The estimated vibrational entropy energy change ($\Delta\Delta S_{Vib}$) and thermal stability (ΔΔG), as calculated from the ENCoM server [5], also indicated an increase of flexibility in p.E155K, p.V297M, p.V316F, p.G433A mutated structures. Extensive MD simulations were carried out for further elucidation, and structural stability of the DSBH core and EF-hand domain were observed during simulations.

Over a simulation period of 100ns, residual fluctuations were retained within 3Å in the EF domain and DSBH core in *wt- P4HTM* (**Figure 1B**), while no domain movement was observed, and structure remained converged (**Figure 1C** and **1D**). For clarity to the reader, these calculations determine the movement of individual residues and can describe areas with greater or lesser movement. Comparison between mutants and wild type can provide insight into how a given mutation affects the local flexibility of the protein; of course, a mutation at a single site can have an impact on the flexibility of apparently distal regions as all components of a protein interact and flexibility is an emergent property. All mutants revealed noticeable Cα-RMSF differences in DSBH core for residues 325 – 355 and 420 – 448 that included the iron binding residues; p.H328 and p.N330 belong to βII, p.H441 belongs to βVII whereas p.E155K also

displayed flexibility in the EF domain (residues 170 – 200) (**Figure 1B**). During the simulation period, the movement was observed in all mutants in βII and βVII, which are involved in the compact architecture of the DSBH core [1]. The substitutions inside the iron binding domain elicited substantial mobility and retained its impact on the entire DSBH core architecture. Overall, mutants revealed fluctuations in both domains and showed domain movements compared to its *wt-P4HTM* (**Figure 1C** and **1D**). The increase in mobility in these functional domains is often associated with a decrease in enzyme functionality.

As P4H domain inhibition protects against obesity and metabolic dysfunction and improves glucose and lipid metabolism[14-16], the mutational consequences in substrate binding site of *P4HTM* were also investigated through docking of HIF-prolyl hydroxylase inhibitor, FG-2216. This enzyme is located at the center of the cell's oxygen-sensing mechanisms, and the active site iron is significantly important in activating oxygen[17]. The *P4H* inhibitors are known to establish an exclusive interaction with Fe^{2+}[1]. The post-MD analysis of FG-2216 revealed slightly different conformations inside the mutants' active site compared to its *wt* conformation (**Figure 1E**). The MMGBSA further explained these conformations based on interaction energetics (**Figure 1F**). Overall, a slight decrease in the binding free energy (ΔG_{tol}) of FG-2216 (p.E155K, -28.11; p.V297M, -30.26; p.V316F, -25.53; G433A, -24.91 kcal/mol) with mutants was expected due to the increased flexibility in their DSBH core region as compared to *wt* (-41.03 kcal/mol). These substitutions eliminated important electrostatic interaction with adjacent residues, leading to deviation and eventually reduced the total binding energy of the complex. This underlying phenomenon might significantly destabilize the substrate binding region of *P4HTM* necessary in activating oxygen and plays a crucial role in the HIF signaling cascade[17].

REFERENCES:

1. Myllykoski, M. *et al.* Structure of transmembrane prolyl 4-hydroxylase reveals unique organization of EF and dioxygenase domains. *Journal of Biological Chemistry* **296**(2021).
2. Dunbrack Jr, R.L. & Karplus, M. Backbone-dependent rotamer library for proteins application to side-chain prediction. *Journal of molecular biology* **230**, 543-574 (1993).
3. Pires, D.E., Ascher, D.B. & Blundell, T.L. DUET: a server for predicting effects of mutations on protein stability using an integrated computational approach. *Nucleic acids research* **42**, W314-W319 (2014).
4. Yan, M. *et al.* Identification of a novel death domain-containing adaptor molecule for ectodysplasin-A receptor that is mutated in crinkled mice. *Current biology* **12**, 409-413 (2002).
5. Frappier, V., Chartier, M. & Najmanovich, R.J. ENCoM server: exploring protein conformational space and the effect of mutations on protein function and stability. *Nucleic acids research* **43**, W395-W400 (2015).
6. Naheed, N. *et al.* New isolate from Salvinia molesta with antioxidant and urease inhibitory activity. *Drug Development Research* **82**, 1169-1181 (2021).
7. Case, D.A. *et al.* Amber 14. (2014).
8. Saeed, S. *et al.* Loss-of-function mutations in ADCY3 cause monogenic severe obesity. *Nature genetics* **50**, 175 (2018).
9. Parveen, A. *et al.* Deleterious variants in WNT10A, EDAR, and EDA causing isolated and syndromic tooth agenesis: A structural perspective from molecular dynamics simulations. *International Journal of Molecular Sciences* **20**, 5282 (2019).
10. Wang, J., Wolf, R.M., Caldwell, J.W., Kollman, P.A. & Case, D.A. Development and testing of a general amber force field. *Journal of computational chemistry* **25**, 1157-1174 (2004).
11. Peters, M.B. *et al.* Structural survey of zinc-containing proteins and development of the zinc AMBER force field (ZAFF). *Journal of chemical theory and computation* **6**, 2935-2947 (2010).
12. Roe, D.R. & Cheatham III, T.E. PTRAJ and CPPTRAJ: software for processing and analysis of molecular dynamics trajectory data. *Journal of chemical theory and computation* **9**, 3084-3095 (2013).
13. Pettersen, E.F. *et al.* UCSF Chimera—a visualization system for exploratory research and analysis. *Journal of computational chemistry* **25**, 1605-1612 (2004).
14. Sugahara, M. *et al.* Prolyl hydroxylase domain inhibitor protects against metabolic disorders and associated kidney disease in obese type 2 diabetic mice. *Journal of the American Society of Nephrology* **31**, 560-577 (2020).
15. Signore, P.E. *et al.* A small-molecule inhibitor of hypoxia-inducible factor prolyl hydroxylase improves obesity, nephropathy and cardiomyopathy in obese ZSF1 rats. *PloS one* **16**, e0255022 (2021).
16. Rahtu-Korpela, L. *et al.* HIF prolyl 4-hydroxylase-2 inhibition improves glucose and lipid metabolism and protects against obesity and metabolic dysfunction. *Diabetes* **63**, 3324-3333 (2014).
17. Poloznikov, A.A. *et al.* Structure–Activity Relationships and Transcriptomic Analysis of Hypoxia-Inducible Factor Prolyl Hydroxylase Inhibitors. *Antioxidants* **11**, 220 (2022).
